# Yellow fever transmission in non-human primates, Bahia, Northeastern Brazil

**DOI:** 10.1371/journal.pntd.0008405

**Published:** 2020-08-11

**Authors:** Jaqueline Goes de Jesus, Tiago Gräf, Marta Giovanetti, Maria Angélica Mares-Guia, Joilson Xavier, Maricelia Lima Maia, Vagner Fonseca, Allison Fabri, Roberto Fonseca dos Santos, Felicidade Mota Pereira, Leandro Ferraz Oliveira Santos, Luciana Reboredo de Oliveira da Silva, Zuinara Pereira Gusmão Maia, Jananci Xavier Gomes Cerqueira, Julien Thèze, Leandro Abade, Mirza de Carvalho Santana Cordeiro, Sintia Sacramento Cerqueira Torquato, Eloisa Bahia Santana, Neuza Santos de Jesus Silva, Rosemary Sarmento Oitiçica Dourado, Ademilson Brás Alves, Adeilde do Socorro Guedes, Pedro Macedo da Silva Filho, Nuno Rodrigues Faria, Carlos F. Campelo de Albuquerque, André Luiz de Abreu, Alessandro Pecego Martins Romano, Julio Croda, Rodrigo Fabiano do Carmo Said, Gabriel Muricy Cunha, Jeane Magnavita da Fonseca Cerqueira, Arabela Leal e Silva de Mello, Ana Maria Bispo de Filippis, Luiz Carlos Junior Alcantara

**Affiliations:** 1 Laboratório de Patologia Experimental, Instituto Gonçalo Moniz, Fundação Oswaldo Cruz, Salvador, Brazil; 2 Laboratório de Parasitologia Médica, Instituto de Medicina Tropical de São Paulo, Universidade de São Paulo, São Paulo, Brazil; 3 Laboratorio de Flavivirus, Fundacao Oswaldo Cruz, Rio de Janeiro, Brazil; 4 Laboratório de Genética Celular e Molecular, ICB, Universidade Federal de Minas Gerais, Belo Horizonte, Minas Gerais, Brazil; 5 Universidade Estadual de Feira de Santana, Feira de Santana, Brazil; 6 Secretaria de Saúde de Feira de Santana, Ministério da Saúde, Feira de Santana, Brazil; 7 KwaZulu-Natal Research Innovation and Sequencing Platform (KRISP), College of Health Sciences, University of KwaZulu-Natal, Durban 4001, South Africa; 8 Laboratório Central de Saúde Pública da Bahia Professor Gonçalo Moniz (LACEN/BA), Salvador, Bahia, Brazil; 9 Faculdade Maria Milza—FAMAM, Bahia, Brazil; 10 Vigilância Epidemiológica do Estado da Bahia, Secretaria de Saúde do Estado da Bahia, Salvador, Brazil; 11 Department of Zoology, University of Oxford, Oxford, United Kingdom; 12 The Global Health Network, Nuffield Department of Medicine, University of Oxford, Oxford, United Kingdom; 13 Organização Pan-Americana da Saúde/Organização Mundial da Saúde, Brasília, Distrito Federal, Brazil; 14 Coordenação Geral dos Laboratórios de Saúde Pública/Secretaria de Vigilância em Saúde, Ministério da Saúde, (CGLAB/SVS-MS) Brasília, Distrito Federal, Brazil; 15 Coordenação Geral de Vigilância de Arboviroses (CGARB); 16 Departamento de Vigilância de Doenças Transmissíveis/Secretaria de Vigilância em Saúde, Ministério da Saúde (DEVIT/SVS-MS); Fundaçao Oswaldo Cruz, BRAZIL

## Abstract

Yellow fever virus (YFV) causes a clinical syndrome of acute hemorrhagic hepatitis. YFV transmission involves non-human primates (NHP), mosquitoes and humans. By late 2016, Brazil experienced the largest YFV outbreak of the last 100 years, with 2050 human confirmed cases, with 681 cases ending in death and 764 confirmed epizootic cases in NHP. Among affected areas, Bahia state in Northeastern was the only region with no autochthonous human cases. By using next generation sequence approach, we investigated the molecular epidemiology of YFV in NHP in Bahia and discuss what factors might have prevented human cases. We investigated 47 YFV positive tissue samples from NHP cases to generate 8 novel YFV genomes. ML phylogenetic tree reconstructions and automated subtyping tools placed the newly generated genomes within the South American genotype I (SA I). Our analysis revealed that the YFV genomes from Bahia formed two distinct well-supported phylogenetic clusters that emerged most likely of an introduction from Minas Gerais and Espírito Santo states. Vegetation coverage analysis performed shows predominantly low to medium vegetation coverage in Bahia state. Together, our findings support the hypothesis of two independent YFV SA-I introductions. We also highlighted the effectiveness of the actions taken by epidemiological surveillance team of the state to prevented human cases.

## Author summary

Between 2016 and 2018, Brazil witnessed the largest YFV outbreak since years, with 2.154 cases and 745 deaths. Here we report the introduction of the virus in the state of Bahia suggesting two independent introductions from Minas Gerais and Espirito Santo. We also highlight the actions taken by epidemiological surveillance division that may have prevent human infections.

## Introduction

Yellow fever virus (YFV) is an enveloped, single-stranded RNA virus, with an approximately 11,000 base pair genome, belonging to the *Flaviviridae* family. It causes a clinical syndrome of acute hemorrhagic hepatitis, called Yellow Fever (YF) disease, that shows clinical features ranging from mild febrile illness to hemorrhage. The term "yellow" comes from jaundice observed in patients with severe liver disease [1–3]. In the Americas, YFV transmission occurs via two distinct cycles: sylvatic and urban. The sylvatic (or jungle) cycle involves transmission between non-human primates (NHP) and sylvatic mosquitoes; humans are infected through the bites of infected sylvatic mosquitoes while visiting or working in forested regions. The urban (or domestic) transmission cycle involves viremic humans who were infected by the virus in the forest regions or through the intermediate cycle, which then returns to an urban area. Those humans develop significant viremia to infect urban mosquitoes which can transmit the virus to other humans in urban settings [4–5]. The main vectors of sylvatic cycle are the *Culicidae* of genus *Haemagogus* and *Sabethes*, which are of strictly wild habits [6–7]. On the other hand, ***Aedes aegypti*** and ***Ae*. *albopictus*** are responsible for outbreaks in urban and peri-urban areas [8–9]. In Brazil, YFV has been reported beyond the limits of Amazon region, occurring throughout the country in cycles of 7 to 14 years [10]. Considering this, Brazilian health authorities adopted surveillance based on seasonality [11]. **Between 2016 and 2018**, Brazil witnessed the largest YFV outbreak since 1980, with 778 cases and 262 deaths during the 2016–2017 seasonal period and 1376 cases and 483 deaths during the 2017–2018 seasonal period [12]. The outbreak has brought national and international concern due to the dispersion of the epidemic in areas where vaccination coverage is limited [13], and due to its potential for international spread [14]. Genomic and epidemiological analysis of the first epidemic wave of the outbreak in Minas Gerais (2016–2017) revealed that human cases resulted from sylvatic transmission [7]. Yet, following the discovery of a pool of ***Ae*. *albopictus*** mosquitoes positive for yellow fever virus, the re-emergence of urban yellow fever has greatly concerned public health authorities [5, 11, 15]. Bahia is the largest state of the Brazilian northeast region, bordering the state of Minas Gerais, the epicentre of the 2016–2017 YFV outbreak in Brazil. Between July 2017 and June 2018, the epidemiological surveillance division reported 58 human YF suspected cases in Bahia, from which laboratory tests confirmed all cases were imported, with no autochthonous confirmed cases in the state. Regarding epizootic cases in NHPs, through June 2017, 624 NHP suspected cases were reported, of which 52 were confirmed by YFV RT-PCR. These cases were widely distributed throughout 28 municipalities in Bahia state, including the capital city, Salvador [16]. In the view of the recent YFV epizootic in Bahia and given the lack of vaccination in the state, it is surprising that no human cases have been reported so far. Considering the seasonality of the virus transmission in the country and the release of epidemiological data reports, from July 2017 to June 2018, 846 NHP epizootic cases were reported, and none was confirmed by laboratory tests. Also, in the period between July 2018 and June 2019, 268 NHP epizootic suspected cases have been reported in 61 municipalities in Bahia state, none was confirmed for YFV infection [16].

In this study, by using the genomic sequence approach, we investigate the molecular epidemiology of YFV in NHP in Bahia and discuss what factors might be acting to prevent virus transmissions to humans [16].

## Materials and methods

### Ethical statement for biological data

This study is part of the arboviral genomic surveillance efforts of the ZiBRA project (www.zibraproject.org) which is supported by the Pan American World Health Organization (PAHO) and the Brazilian Ministry of Health (MoH). Ethical approval for tested samples was obtained from Ethics Review Committee from PAHO under reference number 2016-08-0029. Samples were provided for research and surveillance purposes within the terms of Resolution 510/2016 of CONEP (National Ethical Committee for Research, Ministry of Health, Brazil).

### Population of study

We conducted molecular diagnostics and genome sequencing on 47 YFV positive tissue samples from dead NHPs collected in 24 municipalities in the state of Bahia, Northeast Brazil. Samples were delivered to the Central Public Health Laboratory (LACEN/Bahia) to perform differential diagnosis of rabies and also for collection of viscera samples to investigate YFV infection. Sample data as NHP species, municipality of collection and collection date are summarized in Table 1.

**Table 1:** Epidemiological data associated with each isolate processed/sequenced in this study.

ID	Sample Type	Host	Municipality	Collection Date
RJ250	Liver	*Callithrix* genus	São Miguel das Matas	12/03/2017
RJ251*	Liver	*Callithrix* genus	Cordeiros	10/03/2017
RJ252	Liver	*Callithrix* genus	São Felipe	10/03/2017
RJ253	Liver	*Callithrix* genus	Salvador	14/03/2017
RJ254	Liver	*Callithrix* genus	Feira de Santana	10/03/2017
RJ255	Liver	*Callithrix* genus	Camaçari	14/03/2017
RJ256	Liver	*Callithrix* genus	Alagoinhas	14/03/2017
RJ257	Liver	*Callithrix* genus	Catu	15/03/2017
RJ258*	Liver	no data available	Pedrão	15/03/2017
RJ259*	Liver	*Callithrix* genus	Salvador	15/03/2017
RJ260*	Liver	*Alouatta* genus	Santa Rita de Cassia	13/03/2017
RJ261*	Liver	*Callithrix* genus	Camacari	16/03/2017
RJ262	Liver	*Callithrix* genus	Camacari	16/03/2017
RJ263	Liver	*Callithrix* genus	Camacari	16/03/2017
RJ264	Liver	*Callithrix* genus	Camacari	16/03/2017
RJ265	Liver	*Callithrix* genus	Salvador	16/03/2017
RJ266	Liver	*Callithrix* genus	Ituberá	14/03/2017
RJ267	Liver	*Callithrix* genus	Salvador	19/03/2017
RJ268	Liver	*Callithrix* genus	Feira De Santana	17/03/2017
RJ269*	Liver	*Alouatta* genus	Cordeiros	17/03/2017
RJ270	Liver	*Callithrix penicillata*	Saúde	17/03/2017
RJ271	Liver	*Callithrix jacchus*	Esplanada	21/03/2017
RJ272	Liver	*Callithrix jacchus*	Biritinga	22/03/2017
RJ273	Liver	*Callithrix penicillata*	Barrocas	05/04/2017
RJ274*	Liver	*Callithrix penicillata*	Ichu	05/04/2017
RJ275	Liver	*Callithrix jacchus*	Lauro de Freitas	04/04/2017
RJ276	Liver	*Callithrix jacchus*	Paulo Afonso	05/04/2017
RJ277	Liver	*Callithrix penicillata*	Salvador	06/04/2017
RJ278	Liver	*Callithrix penicillata*	Candeias	06/04/2017
RJ279	Liver	*Callithrix jacchus*	Mata De São Joao	06/04/2017
RJ280	Liver	*Callithrix jacchus*	Salvador	05/04/2017
RJ281	Liver	*Callithrix jacchus*	Salvador	05/04/2017
RJ282	Liver	*Callithrix jacchus*	Salvador	06/04/2017
RJ283	Liver	*Callithrix penicillata*	Salvador	06/04/2017
RJ284	Liver	*Callithrix jacchus*	Salvador	06/04/2017
RJ285	Liver	*Callithrix jacchus*	Salvador	06/04/2017
RJ286	Liver	*Callithrix penicillata*	Camaçari	07/04/2017
RJ287	Liver	*Callithrix penicillata*	Feira de Santana	06/04/2017
RJ288	Liver	*Callithrix penicillata*	Riachão do Jacuipe	07/04/2017
RJ289	Liver	*Callithrix jacchus*	São Goncalo dos Campos	07/04/2017
RJ290	Liver	*Callithrix jacchus*	São Goncalo dos Campos	07/04/2017
RJ291	Liver	*Callithrix penicillata*	Riachao do Jacuipe	07/04/2017
RJ292*	Liver	*Callithrix penicillata*	Feira de Santana	07/04/2017
RJ293	Liver	*Callithrix penicillata*	Feira de Santana	07/04/2017
RJ294	Liver	*Callithrix jacchus*	Salvador	09/04/2017
RJ295	Liver	*Callithrix penicillata*	Irara	07/04/2017
RJ296	Liver	*Callithrix jacchus*	Entre Rios	05/04/2017

ID = Project identifier; Sample Type = Sample type tested; Host = Host species; State: BA = Bahia; Municipality = municipality of sample collection. Collection Date = Date of sample collection. Complete genomes recovered are highlighted with an asterisk.

### Viral RNA isolation and sample processing

Viral RNA was isolated from liver tissue samples using Qiagen Viral RNA Minikit following manufacturer instructions and including additional steps to avoid contamination as previously described by Faria *et al*, 2018 [7]. The first step consisted of homogenization of liver tissue using TissueLyser LT equipment (Qiagen). A ~2 mm diameter of tissue was cut using a disposable scalpel and added to a 2 mL Eppendorf tube containing a 5 mm stainless steel bead (Qiagen). 560 μl AVL lysis buffer (Qiagen) was added to each tube and the sample was homogenised for 5 min at 50 Hz followed by a 10 min incubation at room temperature. Samples were centrifuged at 1,200g for 2 min to pellet cellular material, and 500 μL of supernatant was transferred to a new tube containing 500 μL of 100% EtOH. RNA extraction was subsequently completed using kit. To avoid contamination between samples due to the high number of virions, regular glove changes were conducted and parafilm was used to seal the gap between collection tubes and QIAamp Mini columns (Qiagen) during centrifugation.

### Real-time quantitative PCR

YFV reverse transcription quantitative real-time PCR (RT-qPCR) was performed on 47 samples using the Superscript III Platinum One-Step RT-qPCR System (Invitrogen) on a StepOnePlus Real-Time PCR machine (Applied Biosystems). The conserved YFV 5’ non-coding region was targeted using the primers YFall15F (5’ to 3’: GCTAATTGAGGTGYATTGGTCTGC), YFall103R (5’ to 3’: CTGCTAATCGCTCAAMGAACG) and the probe YFall41 (5’ to 3’: FAM- ATCGAGTTGCTAGGCAATAAACAC-BHQ), based on the previously described Domingo’s assay [17]. Thermocycler conditions consisted of reverse transcription at 45ºC for 15 min, denaturation at 95°C for 5 min, followed by 40 cycles of denaturation at 95°C for 10s, and annealing and extension at 60°C for 40s. To check if RNA isolation was efficient, we used RNase P as an endogenous positive control. Assays for RNase P used the primers RNaseP-F (5’ to 3’: AGATTTGGACCTGCGAGCG), RNaseP-R (GAGCGGCTGTCTCCACAAGT), and a probe (FAM- TTCTGACCTGAAGGCTCTGCGCG-BHQ1).

### cDNA synthesis, library preparation and sequencing for MinION

cDNA was reverse transcribed from viral RNA using the Protoscript II First Strand Sequencing kit (NEB) with random hexamer priming. Multiplex PCR was conducted using Q5 High Fidelity Hot-Start DNA Polymerase (New England Biolabs) and the 500bp sequencing primer scheme as previously described [7]. All samples were subjected to 35 cycles of PCR using the thermocycling conditions and reaction conditions described in Faria *et al*, 2018 [7]. PCR products were purified using a 1x Ampure XP bead clean up and concentrations were measured using a Qubit dsDNA High Sensitivity kit on a Qubit 3.0 fluorimeter (ThermoFisher). Library preparation for the ONT MinION was conducted using Ligation Sequencing 1D (SQK-LSK108) and Native Barcoding kit (EXP-NBD103) according to the manufacturer’s instructions, but with the changes detailed in [18]. Amplified DNA and appropriate negative controls were sequenced in barcoded multiplexes of 6–12 samples per MinION run using FLO-MIN106 flow cells. Sequencing was performed without basecalling for 48 hours using MinKNOW. Consensus sequences for each barcoded sample were generated following previously published methods [18]. Raw files were basecalled using Albacore, demultiplexed and trimmed using Porechop, and then mapped with *bwa* to a reference genome (GenBank Accession No. JF912190). Nanopolish variant calling was applied to the assembly to detect single nucleotide variants to the reference genome. Consensus sequences were generated; non-overlapped primer binding sites, and sites for which coverage was <20X were replaced with ambiguity code N.

### Phylogenetic analysis

YFV sequence dataset was created by adding the eight novel genomes generated in this study to a reference dataset compiled by Faria et al. (2018) [7] and publicly available at https://github.com/arbospread/YFV-monitoring. Sequences were aligned using MAFFT [19] and manually edited using AliView [20]. Maximum likelihood (ML) phylogenies were performed in IQ-TREE program under a GTR + Γ_4_ nucleotide substitution model. [21]. Prior to the tree reconstruction, ModelFinder application [22], as implemented in IQ-TREE, was used to select the best-fitted substitution model for the analysed dataset. Time-scaled phylogenetic trees were inferred with the sequences clustering in the current YFV outbreak clade. Temporal signal was assessed in TempEst [23] and the BEAST/BEAGLE package [24] was used for the Bayesian phylogenetic analysis. Trees were estimated under the SRD06 substitution model [25], the uncorrelated lognormal distributed relaxed molecular clock [26] and the nonparametric Bayesian Skygrid coalescent model [27]. Monte Carlo Markov chains (MCMC) were run for 100 million generations and the chain convergence and adequate effective sample size (ESS) were diagnosed in Tracer software [28]. Maximum clade credibility (MCC) trees were summarized using the TreeAnnotator tool and visualized in Figtree v1.4.4 (https://github.com/rambaut/figtree).

## Results

### Sample Collection, RT-qPCR screening and sequencing

**Fig 1:**
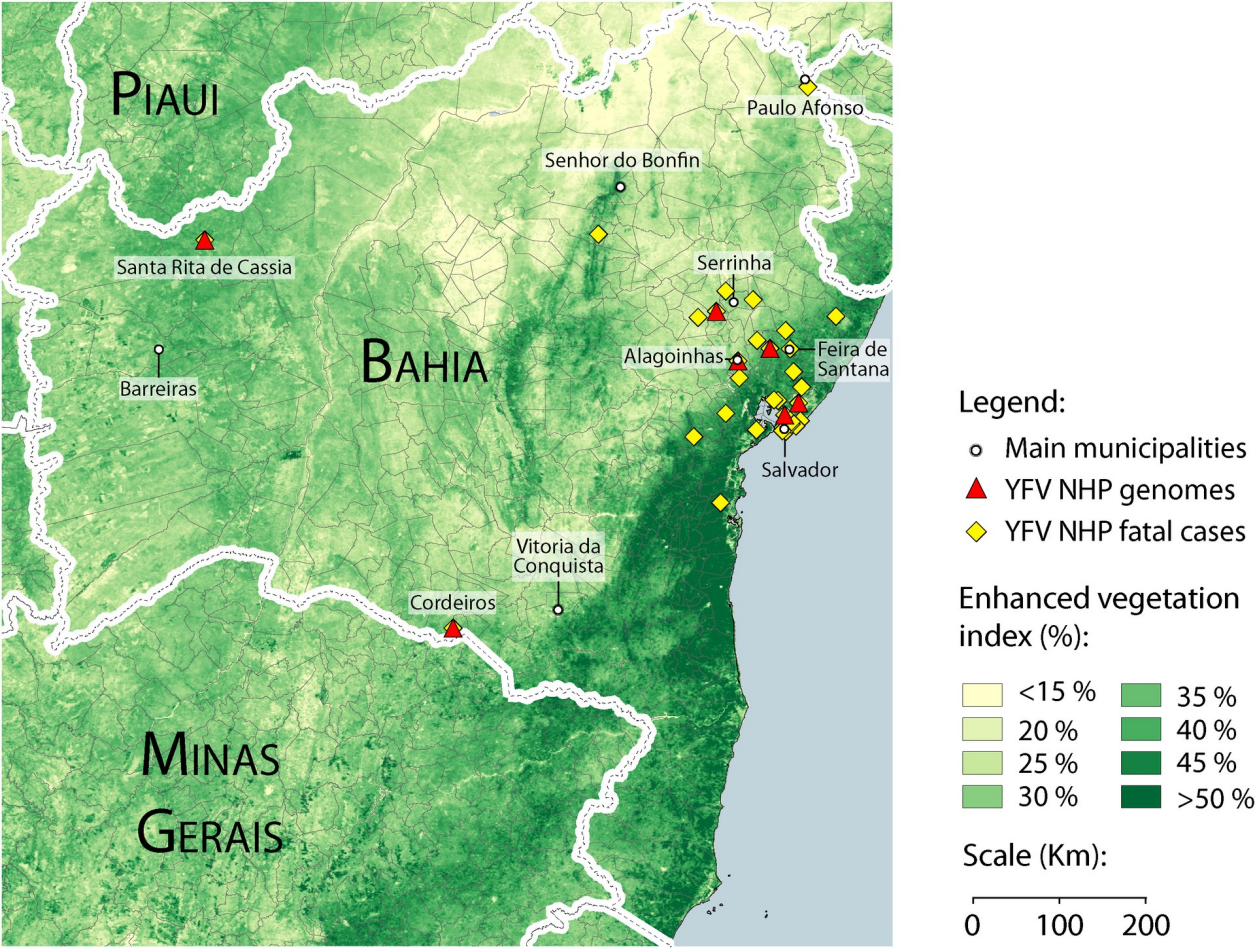
Geographical distribution of YFV NHP cases in Bahia state and vegetation coverage analysis. Sampling locations of YFV genome sequences generated in this study are shown as red triangles. Main municipalities are represented as white circles. Yellow diamonds show all epizootic cases tested on the study. Green color gradient shows vegetation index of Bahia state.

We conducted molecular screening of YFV on liver fragments of non-human primates collected in 24 municipalities of the Bahia state, northeastern Brazil. Most of these municipalities are concentrated in the eastern region of the state, in the vicinity of the cities of Salvador and Feira de Santana (Fig 1). However, some samples were collected in more distant regions, especially those collected in the northwest of the state, in the city of Santa Rita de Cássia. Additionally, four samples are from the southern city of Vitória da Conquista. Among the tested samples, non-human primates from the *Callitrichidae* family (n = 44) predominated (*Callithrix* genus: *Callithrix jacchus*, n = 14; *Callithrix penicillata* n = 13), but we also included non-human primates from the *Atelidae* family (*Alouatta* genus n = 2) (Table 1). Due to the deteriorated conditions of the primates, 17 *Callithrix* could only be identified at the species level by the surveillance team. YFV RT-qPCR tests results, expressed as cycle threshold values (Ct), ranged from 7.59 to 36.81 (median = 31.31) (Table 1). The majority of samples had Ct > 30.0, all from *Callithrix* genus.

### Genome sequencing and phylogenetic analysis

Genomes were generated using a previously validated MinION portable YFV sequencing protocol [7]. Briefly, positive samples were enriched by performing a multiplex PCR, using two sets of overlapping primers to amplify the YFV complete genome. Samples with amplicon yield from 4.0 ng/uL and above were sequenced. Due to high Ct values ​​and, consequently, low viral load, it was possible to recover eight complete genomes, with genome coverage (20x) similar for all samples (median = 99%; S1 Table). ML phylogenetic tree reconstructions and automated subtyping tools (Yellow Fever Virus Typingtool available at www.genomedetective.com/app/typingtool/yellowfevervirus/) [7] placed the newly generated genomes within the South American genotype I, among the sequences of the current Brazilian outbreak (Fig 1).

**Fig 2:**
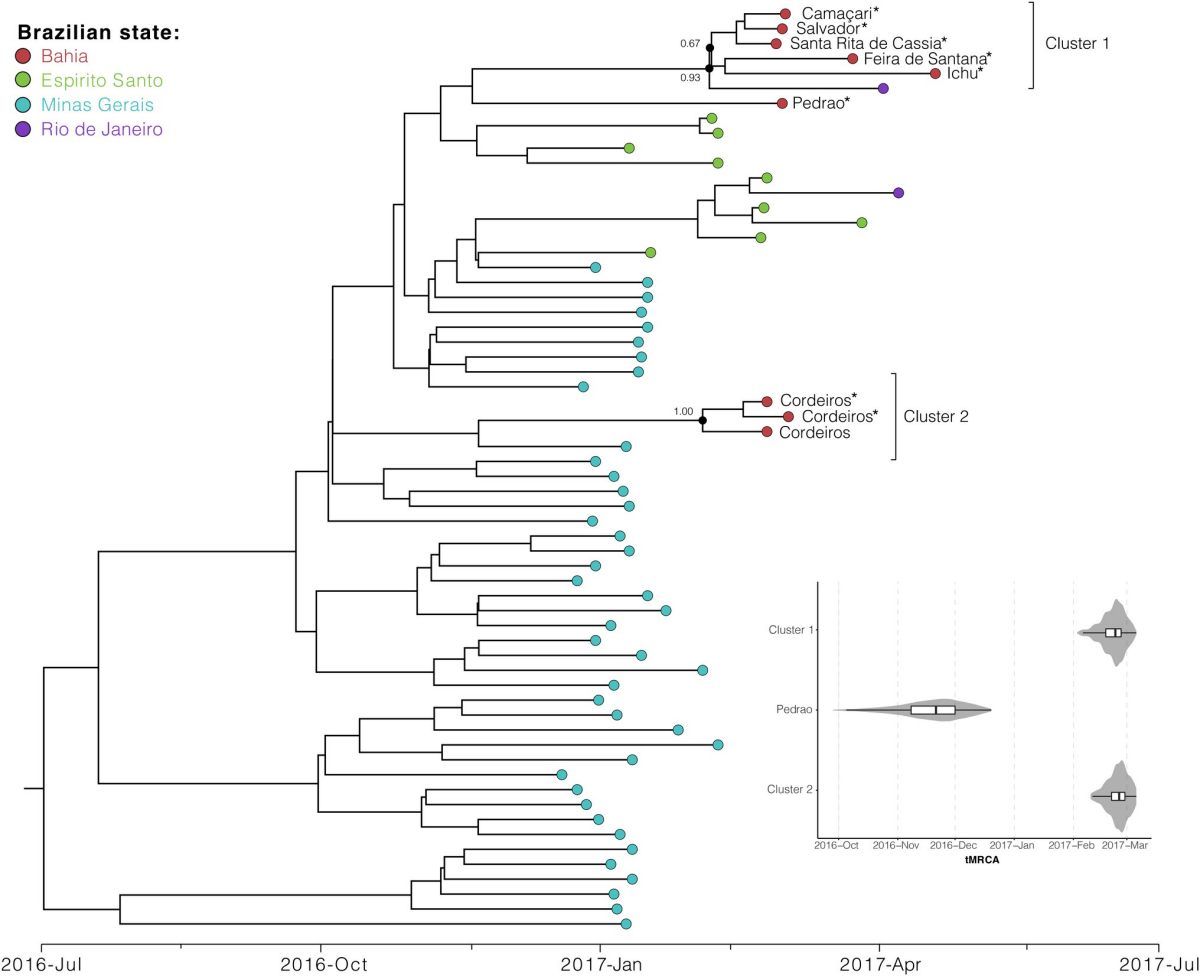
Bayesian and molecular phylogenetic of the YFV NHP cases in Bahia. Bayesian dated phylogenetic analysis revealed that the YFV genomes isolated from NHP infected in Bahia formed two distinct well-supported phylogenetic clusters. Cluster 1 (red colored superior sequences; posterior probability, PP = 0.93) emerged most likely from an introduction from Minas Gerais state (red colored inferior sequences PP = 0.67), however an origin in Espírito Santo state is also possible. Cluster 2 (PP = 1.00) originated from an introduction from Minas Gerais (PP = 1.00) composed by three sequences isolated from NHPs sampled in the city of Cordeiros, located further south in the Bahia state and close to the Minas Gerais state borders. Violin plots showing estimated posterior distributions of the time of the most recent common ancestor (tMRCA). The time of the most recent common ancestor of both clusters is late February 2017 (95% Bayesian credible interval: February to March 2017).

Our Bayesian dated phylogenetic analysis revealed that the YFV genomes isolated from NHP infected in Bahia formed two distinct well-supported phylogenetic clusters. Cluster 1 is composed by sequences isolated from infected NHP found in the municipalities of Camaçari, Salvador, Feira de Santana and Ichu, which are separated of a maximum great-circle distance of 156 km. Cluster 1 is also composed of a sequence isolated in the city of Santa Rita de Cassia, which is at a minimal distance of 585 km (great-circle distance) apart from the other cities in the same cluster. A single sequence from the municipality of Pedrao is located basally to Cluster 1, however with small support to form a clade with those sequences. Within Cluster 1, was also observed a YFV genome isolated in Rio de Janeiro state and basally to the whole cluster, sequences from Espirito Santo state are located. This, suggests that Cluster 1 has emerged from an introduction from one of these two states, however, only future studies with higher sampling coverage from Bahia state could provide more information about the origins and transmission dynamics of YFV in northeast Brazil. In contrast, our phylogenetic analysis shows that Cluster 2 (pp = 1.00), composed by three sequences isolated from NHPs sampled in the city of Cordeiros, suggests that Minas Gerais state is the origin of this cluster. Cordeiros is located further south in the Bahia state and close to the Minas Gerais state borders. The time of the most recent common ancestor of both clusters (Fig 2) is late February 2017 (95% Bayesian credible interval: February to March 2017).

## Discussion

In the last two decades, YFV transmissions have been recorded beyond the limits of the considered endemic area (Amazon region). Human cases and/or NHP epizootic cases occurring in Bahia, Minas Gerais, São Paulo, Paraná and Rio Grande do Sul characterize a recurrent expansion of the viral circulation area in the eastern and south directions of the country, where the virus had not been registered for decades [29].

One of the most significant events in the history of YF was recorded in the 2017/2018 period: the spread of the virus reached the Brazilian east coast, in the region of the Atlantic Forest biome, which houses a wide diversity of non-human primates and potential wild vectors [7, 13, 15].

This recent YFV outbreak has been favored by changes in environmental, eco-social and human behavior [30]. Although Bahia state did not report autochthonous human cases, a large number of epizootic cases in NHP have been reported in that state [16].

Here we reported the generation of novel YFV sequences recovered from NHP from the northeast region of Brazil not covered before by other studies. Our phylogenetic analysis has shown the simultaneous introduction of YFV into two distinct sites in the Bahia state. Considering the distance between the locations, these introductions are consistent with the hypothesis that animals may have been transported by humans as some events of primate’s traffic by human activity has already been reported by surveillance teams within Bahia state (data not published). Possas and colleagues [31] discussed that NHPs are not responsible for the rapid spread of YFV observed in the last outbreak in Brazil, once the migration habits of NHP species in Brazilian Southeastern forests show evidences that they do not use the ground and deforested areas to migrate.

According to our vegetation coverage analysis, Bahia state shows predominantly low to medium vegetation coverage (Fig 1). In addition to our knowledge about mosquitoes’ speed mobility and the time between the two introductions we suggest that the occurrence of cases in distant areas was not caused by NHP natural migration nor by mosquitoes spread from an initial point [7].

Factors like (i) high density of vectors and primary hosts, (ii) the presence of susceptible individuals and (iii) low vaccine coverage areas play an important role in the emergence of a YFV outbreak. Over the last decades YF cases have been controlled in Brazil through the efforts of the epidemiological surveillance teams that acts in a preventive way, through investigation and deployment of vector barriers in places considered at risk of transmission, avoiding the spread of the virus to other regions and also the re-establishment of urban transmission [16].

Upon the resurgence of YF in southeastern, Bahia state has intensified the vaccine campaign and it was widely distributed to population in municipalities bordering states with YFV circulation or where there was NHP YFV detection.

According to the vaccination recommendation area, the municipalities covered by intensification of campaign in 2018 were Camaçari, Candeias, Itaparica, Lauro de Freitas, Mata de São João, Salvador, São Francisco do Conde and Vera Cruz. Still according to Brazilian surveillance epidemiological reports, between July 2017 and June 2018, the state reported 378 cases of NHP YFV, still lower than the Southeastern states of Sao Paulo (2,119 reported cases), Minas Gerais (n = 1,600) and Rio de Janeiro (n = 622). [32].

The 2016–2018 YFV outbreak in Brazil has shown human cases with deaths in the south-eastern states, especially Minas Gerais, where the outbreak began and has expanded. Considering the proximity between the state of Minas Gerais and the state of Bahia, together with the wide circulation of the vector, the occurrence of NHP infected by the YFV and low vaccine coverage in Bahia state, we wondered why Bahia did not present the occurrence of human cases.

The best hypothesis to explain this fact lies in two main aspects: the first is related to the type of NHP infected by the virus, predominantly *Callithrichidae* family primates, which, as described by other studies, have a lower viral replication ability, reducing its transmission through the sylvatic cycle [5, 33–34].

The goal of monitoring epizootic cases among NHP is to provide an early warning of risk of YFV transmission to humans, the second fact is based on the actions of epidemiological surveillance teams (municipal and of the state) that together carried out several activities to reduce virus transmission. Beyond vaccination campaign, vector blockade with insecticide (heavy and costly ultra-low volume application) which is already part of the strategies used by the epidemiological surveillance service in Brazil and was intensified in the state of Bahia. The use of agrochemicals in public health is standardized by the WHO as a complementary tool for vector control and it was adopted by the Health Surveillance Secretariat of the Ministry of Health in Brazil [35]. In addition, the investigation of suspicious cases in a timely manner was crucial to the prevention of urban yellow fever favored by *Ae*. *Aegypti* vector. The integrated actions promoted a blockade in the transmission chain of 28 municipalities with laboratory proven NHP YFV circulation, so that there would be no record of a human case with autochthonous transmission in Bahia between December/2016 and June/2019. During the monitoring period (July/2018 to June/2019), 216 NHP cases were reported in 61 municipalities. Considering this post-outbreak epidemiological data, when compared with the previous monitoring period (July/2017 to June/2018), with a record of 846 cases, a reduction of 291% compared to the current period. Thus, the coordinated actions of epidemiological surveillance team in Bahia state were the main factor for preventing human cases during the YFV epidemics which affected other states in Brazil. Vector surveillance and control are essential for the prevention and control of vector borne diseases and, in YF epidemics, the identification of *Ae*. *aegypti* and other *Ae*. species helps to inform authorities the level of risk of an urban outbreak [35]. In Bahia state, epidemiological surveillance actions such as the use of vaccine blocking strategies and vector control possibly contributed to the success in preventing autochthonous human YFV cases during the 2016–2018 epidemics in Brazil and should be taken as an example of coordinated management for future vector borne diseases outbreaks.

## Conclusion

Together, our findings support the hypothesis of two independent YFV SA-I introductions and highlighted the possible contribution of the actions taken by epidemiological surveillance team of the state of Bahia on prevention of human cases.

## Supporting information

S1 Table Statistics for the YFV sequences generated in this study. Sequences were mapped against FJ912190 reference genome. Numbers correspond quantity of reads mapped on reference genome. Ct = RT-qPCR Cycle threshold value. Accession number correspond to Genbank database accession number.
(DOCX)

## References

[1] Chen C, Jiang D, Ni Met al. Phylogenomic analysis unravels evolution of yellow fever virus within hosts. PLOS Neglected Tropical Diseases, 2018;12(9) e0006738 10.1371/journal.pntd.0006738 30188905 PMC6143276

[2] Sanna A, Andrieu A, Carvalho L et al Yellow fever cases in French Guiana, evidence of an active circulation in the Guiana Shield, 2017 and 2018. Euro Surveillance: Bulletin Europeen Sur Les Maladies Transmissibles = European Communicable Disease Bulletin, 2018; 23(36). 10.2807/1560-7917.ES.2018.23.36.1800471 PMC6134805 30205871

[3] Simon LV, Hashmi MF, Torp KD. Yellow Fever. [Updated 2019 Sep 29]. In: StatPearls [Internet]. Treasure Island (FL): StatPearls Publishing; 2019. 29262028

[4] Huang YJS, Nuckols JT, Horne KM et al Mutagenesis analysis of T380R mutation in the envelope protein of yellow fever virus. Virology Journal, 2014;11(1):60. 24678844 10.1186/1743-422X-11-60 PMC3974419

[5] Possas C, Martins RM, Oliveira RL et al Urgent call for action: avoiding spread and re-urbanisation of yellow fever in Brazil. Memórias Do Instituto Oswaldo Cruz. 2018;113:1–2. 10.1590/0074-02760170361 29185597 PMC5719535

[6] Cardoso JC, de Almeida MAB, dos Santos E et al Yellow fever virus in Haemagogus leucocelaenus and Aedes serratus mosquitoes, southern Brazil, 2008. Emerging Infectious Diseases. 2010;16(12):1918–1924. 10.3201/eid1612.100608 21122222 PMC3294583

[7] Faria NR, Kraemer MUG, Hill SC et al Genomic and epidemiological monitoring of yellow fever virus transmission potential. Science. 2018;361(6405):894–899. 10.1126/science.aat7115 30139911 PMC6874500

[8] Mondet B. [Epidemiology of arbovirus diseases: use and value of physiologic age determination of female mosquito vectors]. Bulletin de La Société de Pathologie Exotique. 1996;89(2):155–60. French 8924776

[9] Mondet B. [Yellow fever epidemiology in Brazil]. Bulletin de la Societe de pathologie exotique. 2001;94(3):260–267.French 11681224

[10] Câmara FP, Gomes ALBB, Carvalho LM F et al Dynamic behavior of sylvatic yellow fever in Brazil (1954–2008). Revista Da Sociedade Brasileira de Medicina Tropical. 2011;44:297–299. 10.1590/s0037-86822011005000024 21537794

[11] Brasil, Ministério da Saúde, Secretaria de Vigilância em Saúde. Boletim Epidemiológico: Monitoramento de Febre Amarela Brasil 2019(a), informe nº18, 9 jun 2019.

[12] World Health Organization. Emergencies preparedness, response—Yellow fever–Brazil. 18Apr2019, 2019. Available on https://www.who.int/csr/don/18-april-2019-yellow-fever-brazil/en/.

[13] Brasil, Ministério da Saúde, Secretaria de Vigilância em Saúde. Boletim Epidemiológico: Monitoramento dos casos de dengue, febre de chikungunya e febre pelo vírus Zika até a Semana Epidemiológica 6, 2016. v. 47, n. 10, p. 7, 2016. ISSN 2358-9450. Available on http://portalarquivos2.saude.gov.br/images/pdf/2016/marco/23/2016-007—Dengue-SE-6-publica—-o.pdf

[14] Barrett ADT. The reemergence of yellow fever. Science 2018;361(6405):847–848. 10.1126/science.aau8225 30139914

[15] Brasil, Ministério da Saúde, Secretaria de Vigilância em Saúde. Boletim Epidemiológico: Monitoramento dos casos de arboviroses urbanas transmitidas pelo Aedes (dengue, chikungunya e Zika) até a Semana Epidemiológica 12 de 2019(b) e Levantamento Rápido de Índices para Aedes aegypti (LIRAa). v. 50, nº 13, ABRIL 2019.

[16] Brasil, Ministério da Saúde, Secretaria de Vigilância em Saúde. Diretoria de Vigilância Epidemiológica. Superintendência de Vigilância em Saúde. Boletim Epidemiológico de Febre Amarela, Bahia, 2019(c). Período de Monitoramento Jul/2018 –Jun/2019.

[17] Domingo C, Patel P, Yillah J et al Advanced Yellow Fever Virus Genome Detection in Point-of-Care Facilities and Reference Laboratories. Journal of Clinical Microbiology, 2012;50(12):4054–4060. 10.1128/JCM.01799-12 23052311 PMC3503008

[18] Quick J, Grubaugh ND, Pullan ST, Claro IM, Smith AD, Gangavarapu K, et al Multiplex PCR method for MinION and Illumina sequencing of Zika and other virus genomes directly from clinical samples. Nature protocols. 2017;12:1261–1276. 10.1038/nprot.2017.066 28538739 PMC5902022

[19] Katoh K, Rozewicki J, Yamada KD. MAFFT online service: multiple sequence alignment, interactive sequence choice and visualization. Briefings in bioinformatics. 2019;20:1160–1166. 10.1093/bib/bbx108 28968734 PMC6781576

[20] Larsson A. AliView: a fast and lightweight alignment viewer and editor for large datasets. Bioinformatics (Oxford, England). 2014;30:3276–3278. 10.1093/bioinformatics/btu531 PMC4221126 25095880

[21] Nguyen LT, Schmidt HA, von Haeseler A et al IQ-TREE: a fast and effective stochastic algorithm for estimating maximum-likelihood phylogenies. Molecular Biology and Evolution.2015;32(1):268–274. 10.1093/molbev/msu300 25371430 PMC4271533

[22] Kalyaanamoorthy S, Minh BQ, Wong TKF et al ModelFinder: fast model selection for accurate phylogenetic estimates. Nature Methods. 2017;14(6):587–589. 10.1038/nmeth.4285 28481363 PMC5453245

[23] Rambaut A, Lam TT, Carvalho LM et al Exploring the temporal structure of heterochronous sequences using TempEst (formerly Path-O-Gen). Virus Evolution. 2016;2(1). 10.1093/ve/vew007 PMC4989882 27774300

[24] Suchard MA, Lemey P, Baele G et al Bayesian phylogenetic and phylodynamic data integration using BEAST 1.10. Virus Evolution. 2018;4(1. 10.1093/ve/vey016 PMC6007674 29942656

[25] Shapiro B, Rambaut A, Drummond AJ.Choosing Appropriate Substitution Models for the Phylogenetic Analysis of Protein-Coding Sequences. Molecular Biology and Evolution. 2005;23(1):7–9. 10.1093/molbev/msj021 16177232

[26] Drummond AJ, Ho SYW, Phillips MJ et al Relaxed Phylogenetics and Dating with Confidence. PLOS Biology. 2006;4(5):e88 10.1371/journal.pbio.0040088 16683862 PMC1395354

[27] Gill MS, Lemey P, Faria NR et al Improving Bayesian Population Dynamics Inference: A Coalescent-Based Model for Multiple Loci. Molecular Biology and Evolution. 2012;30(3):713–724. 10.1093/molbev/mss265 23180580 PMC3563973

[28] Rambaut A, Drummond AJ, Xie D et al Posterior Summarization in Bayesian Phylogenetics Using Tracer 1.7. Systematic Biology. 2018;67(5):901–904. 10.1093/sysbio/syy032 29718447 PMC6101584

[29] BRASIL, Ministério da Saúde, Secretaria de Vigilância em Saúde. Boletim Epidemiológico: Emergência epidemiológica de febre amarela no Brasil, no período de dezembro de 2016 a julho de 2017. v. 48, nº 28, 2017.

[30] Possas C, Lourenço-de-Oliveira R, Tauil PL et al Yellow fever outbreak in Brazil: the puzzle of rapid viral spread and challenges for immunisation. Memórias Do Instituto Oswaldo Cruz. 2018;113(10):e180278 10.1590/0074-02760180278 30427974 PMC6135548

[31] Possas C, Antunes AMS, Mendes FML et al Emerging and Resurgent Arboviral Diseases: Global Vaccine Patent Landscape and the Case for Immunome. In: Singh H., Keswani C., Singh S. (eds) Intellectual Property Issues in Microbiology. Springer, Singapore 2019.

[32] BRASIL, Ministério da Saúde, Secretaria de Vigilância em Saúde. Boletim Epidemiológico: Monitoramento do Período Sazonal da Febre Amarela. Brasil– 2017/2018. Informe nº 27, 2017/2018.

[33] Mares-Guia MAM, Horta MA, Romano A et al Yellow fever epizootics in non-human primates, Southeast and Northeast Brazil (2017 and 2018). Parasites Vectors 2020; 13:90. 10.1186/s13071-020-3966-x PMC7031979 32075684

[34] Almeida MAB, Cardoso JC, dos Santos E et al Surveillance for Yellow Fever Virus in Non-Human Primates in Southern Brazil, 2001–2011: A Tool for Prioritizing Human Populations for Vaccination. PLOS Neglected Tropical Diseases 2014;8(3):e2741 10.1371/journal.pntd.0002741 24625681 PMC3953010

[35] WHO. A global strategy to Eliminate Yellow fever Epidemics 2017–2026. Geneva: World Health Organization

