# Supplementary material for: Yellow fever transmission in non-human primates, Bahia, Northeastern Brazil
Source: PLoS Negl Trop Dis. 2020 Aug 11;14(8):e0008405. doi: 10.1371/journal.pntd.0008405 (PMC7418952; doi:10.1371/journal.pntd.0008405)
Supplement: S1 Table — Sequences were mapped against FJ912190 reference genome. Numbers correspond quantity of reads mapped on reference genome. Ct = RT-qPCR Cycle threshold value. Accession number correspond to Genbank database accession number. (DOCX) [file pntd.0008405.s001.docx]

**Yellow fever transmission in non-human primates, Bahia, Northeastern Brazil**

**S1 Table. Statistics for the YFV sequences generated in this study**

| **Table S1. Statistics for the YFV sequences generated in this study** | | | | |
| --- | --- | --- | --- | --- |
| **Samples** | **Number of reads basecalled/run** | **Coverage against FJ912190 (%)** | **Ct value** | **Acession number** |
| RJ251 | 1,352,071 | 99.67 | 7,59 | MN604280 |
| RJ258 | 3,147,936 | 98.45 | 25,81 | MN604284 |
| RJ259 | 2,280,272 | 96.280 | 30,51 | MN604283 |
| RJ260 | 2,280,272 | 96.278 | 27,43 | MN604281 |
| RJ261 | 2,280,272 | 96.268 | 29,39 | MN604282 |
| RJ269 | 1,352,071 | 97.63 | 8,29 | MN604287 |
| RJ274 | 3,147,936 | 99.85 | 30,68 | MN604285 |
| RJ292 | 3,147,936 | 99.34 | 30,87 | MN604286 |

Sequences were mapped against FJ912190 reference genome. Numbers correspond quantity of reads mapped on reference genome. Ct = RT-qPCR Cycle threshold value. Accession number correspond to Genbank database accession number.
